# Assessing changes in social determinants of health inequalities in South Africa : a decomposition analysis

**DOI:** 10.1186/s12939-018-0885-y

**Published:** 2018-12-11

**Authors:** Kehinde O. Omotoso, Steven F. Koch

**Affiliations:** Department of Economics, University of Pretoria, Private Bag X20, Hatfield 0028, Pretoria, South Africa

**Keywords:** Decomposition, Health inequality, Self-assessed health, Social determinants, South Africa

## Abstract

**Background:**

Despite various policy interventions that have targeted reductions in socio-economic inequalities in health and health care in post-Apartheid South Africa, evidence suggests that not much has really changed. In particular, health inequalities, which are strongly linked to social determinants of health (SDH), persist. This study, thus, examines how changes in the SDH have impacted health inequalities over the last decade, the second since the end of Apartheid.

**Methods:**

Data come from information collected on social determinants of health (SDH) and on health status in the 2004, 2010 and 2014 questionnaires of the South African General Household Surveys (GHSs). The health indicators considered include ill-health status and disability. Concentration indices and Oaxaca-Blinder decomposition of change in a concentration index methods were employed to unravel changes in socio-economic health inequalities and their key social drivers over the studied time period.

**Results:**

The results show that inequalities in ill-health are consistently explained by socio-economic inequalities relating to employment status and provincial differences, which narrowed considerably over the studied periods. Relatedly, disability inequalities are largely explained by shrinking socio-economic inequalities relating to racial groups, educational attainment and provincial differences.

**Conclusion:**

The extent of employment, location and education inequalities suggests the need for improved health care management and further delivery of education and job opportunities; greater effort in this regard is likely to be more beneficial in some way.

## Background

Globally, policymakers and researchers have long sought not only to improve overall population health and reduce burden of disease, ill-health and disability, but also to reduce or eliminate health differentials, based on gender, geography, race, ethnicity and socio-economic status, amongst others [1–7]. To this effect, various governments, at different capacities, have enacted policies and reforms to tackle health inequality and its social determinants [8]. Despite considerable attention on socio-economic related health inequalities, striking differences in health still exist within and among nations, today. Socio-economic related differences within countries are often substantial [9–15], especially in developing African countries [16–19].

### Context

Prior to 1994, South Africa’s health system was divided along racial lines - one system was highly resourced and benefited the white minority, while the other was systematically under-resourced and was for the black majority [20, 21]. Notably, the health system was highly characterised by racial segregation and systemic fragmentation, with 14 separate health departments. Health services were focused on the hospital sector, primary health care services were underdeveloped while health services were systematically underfunded among the black population group [21].

Post 1994, the South African health system has developed into a two-tiered system which is unified and more co-ordinated. The National health plan, developed by the African National Congress (ANC) in 1994, charted a new path for a codified and a more coherent health system [22]. Primary health care (PHC) was made the cornerstone of health policy, with the introduction of free medical treatment for pregnant women and children younger than 6 years in 1994 and 1996, and subsequent extension to all users. The 14 fragmented health departments were also consolidated into one national and nine provincial health departments. Driven by the need to redress inequities and discriminations, the health system is being transformed, through different documented reforms, polices and programmes (including the on-going implementation of the national health insurance (NHI) scheme) [21, 23–31], into an integrated and comprehensive health care system.

The health care system, which provides services for an estimated population of over 58 million people, comprises the private and public sectors. Basically, South Africa’s public health care system contains five layers, namely: primary health care (clinics), district hospitals, regional hospitals, tertiary/academic hospitals and central/academic hospitals. This structure was developed to address cost effective health care for all citizens, on an appropriate level, and to ensure better health for all. The majority of the population access health services through government-run and funded public clinics and hospitals. The public health services are divided into primary, secondary and tertiary through health facilities that are located in, and managed by, the provincial departments of health [32].

South Africa is committed to the health of her citizens and equitable access to better health care services [33]. This right to health is deeply rooted in post-apartheid South Africa’s constitution, which specifies that ‘everyone has the right to have access to health care services, including reproductive health care’ [34]. Thus, everyone has access to both the public and private health services. However, access to private health services depends largely on an individual’s ability to pay: individuals can either choose to pay out-of-pocket or purchase private insurance in order to be treated at private hospitals and health clinics. Public health care provides mostly free services and care to all citizens, including pharmaceuticals, wheelchairs and crutches, and home care visits, amongst others. However, public health care is often characterised by problems, such as long waiting times, rushed appointments, old facilities, poor disease control and prevention practices, and poor quality of care, when compared to private health care [32, 35].

In post-apartheid South Africa, improving health outcomes is prioritised in health and cognate policies and reforms. At the core of these policies and reforms is the continuous fight against the quadruple burden of disease, namely HIV/AIDS and TB, maternal, infant and child mortality, non-communicable/chronic diseases, ill-health/injury and violence that plague the country [36, 37]. Although enormous effort has gone into addressing each of this burden of disease, it remains a challenge to the public health system [29]. This burden of disease is closely associated with the vast socio-economic inequalities that characterise the country, known as one of the most unequal in the world [38–40], even as the government fights against social injustice and socio-economic inequalities in all facets of life. Thus, addressing social determinants of health has been a cornerstone in primary health care (PHC), its re-engineering strategy [41], and the National Development Plan (NDP).

### Policy context

Since the emergence of democracy in the last two decades in South Africa, considerable effort has gone into redressing the damaging impacts of Apartheid, which was characterised by legislated inequality. Specifically, the South African government has embarked on a variety of policies and reforms to reverse the discriminatory practices that pervaded all aspects of life before 1994 [42]. Policy interventions have targeted reductions in socio-economic inequalities in various capacities, and, by extension, these policies have also applied to the health care system: fiscal redistribution targeted at health, education, social protection sectors; abolition of user fees at the primary health care (PHC) level in 1994; extension of PHC policy to all users in relatively poorer households in 1996; introduction of Government Employees MedicalAid Scheme (GEMS) in 2006 and ongoing discussions related to universal health care coverage through yet-to-be-fully-implemented national health insurance (NHI) [23–25, 28, 43, 44]. However, evidence suggests that not much has really changed [32, 45–47]. In particular, health inequalities, which are strongly linked to social determinants of health (SDH), persist [48–50]. To further address socio-economic related health inequalities, a broader understanding of the changes that have occurred over the post-apartheid period might be insightful.

### Problem statement

In the literature, health inequality and its social determinants have received considerable attention [18, 21, 23, 33, 35, 48, 50–58]. Some of the preceding studies find poor self-reported health to be higher among lower socio-economic groups [50, 51, 55]. One study finds that the burdens of major categories of self-reported illness and disability are greater among lower socio-economic groups [50]. In a related study, social protection and employment, knowledge and education, housing and infrastructure contribute to disparities in good self assessed health (SAH) [48]. Moreover, there is strong indirect racial aspect to health inequalities in South Africa [57].

However, the existing empirical analysis of health inequality is, to a large extent, cross-sectional. It focuses at a given point in time and might apply one-way decomposition to examine the contributions of socio-economic factors to health inequality. Moreover, they tend to focus on only a few health indicators. With few indicators, we may only see narrow view of health inequalities and, thus, may underestimate overall health inequality. Single-time assessment of health inequality, on the other hand, may downplay the effects of health inequality-focused reforms, as it does not uncover dynamics that are vital for indirect assessment of the effectiveness, or otherwise, of prior policies and health interventions aimed at reducing socio-economic related health inequalities. For instance, one recent study with cross-sectional one-way decomposition explains the social factors that account for health disparities [48]. It is relevant for understanding health inequalities in South Africa, but only provides information about health inequality at a given point in time. It cannot uncover changes in health inequalities. Yet, it is important to understand those changes and the extent to which they have been impacted by, for example, changes in social factors, especially in a country such as South Africa, which has worked and is still working to redress socio-economic related health inequalities by targeting key social factors. Doing so can help to identify key drivers of changes in health inequality and, thus, improve the efficiency of resource allocations to further reduce health inequalities. Moreover, areas that need further improvement or interventions can be highlighted. It can also serve as feedback during the policy review process, especially when considering reforms directed at socio-economic factors that are often targeted by policy.

To consider these dynamic aspects, we make use of existing methodological developments in the literature to extend previous analyses [48, 50], which we discuss below. The data used in our analysis was sourced from the 2004, 2010 and 2014 General Household Surveys (GHSs). The initial year, 2004 marks the recommendation for the implementation of National Health Insurance in South Africa [44] and coincides with the 10 year anniversary of the end of Apartheid; 2014 marks an additional decade down the line, while 2010 indicates the enactment of key policies, programmes and service priorities to reduce the burden of premature death and improve equity, efficiency and quality of health care in the South African health system [43]. Therefore, the analysis allows us to correlate health policies from the second decade post-apartheid, indirectly, with either a widening or narrowing of health inequality.

Our empirical strategy adopts the concentration index regression model and Oaxaca-type decomposition of change in the concentration index [59, 60]. The concentration index method was employed to uncover the relative change in health inequalities over the studied periods, linking those changes to changes in the SDH. The decomposition of change in the concentration index explains how changes in health inequalities are attributable to changes in social determinants. We apply the method to ill-health status and disability.

## Methods

### Data source

Data used in this study was sourced from three waves of South African General Household Surveys (GHSs); one from 2004 [61], another from 2010 [62] and the other from 2014 [63]. Although GHS data exist for 2002 and 2003, the 2004 survey was chosen because 2004 marks the beginning of the second decade of democracy, while 2014 represents the end of that decade. Although a five-year step would be more obvious, we decide to use 2010 because the survey questions were consistent with the other two surveys, which was not the case with 2009. The GHSs are repeated cross-sectional household surveys collected annually by the national statistical agency, Statistics South Africa (StatsSA), with new samples drawn each year. Each year, the sample includes approximately 30,000 households, following a multi-stage stratified design, such that, each sample is representative at both the national and provincial levels within any year. The surveys collect a range of demographic and socio-economic information on households and individuals across the country’s nine provinces. Survey questions relate to housing services, social services, socio-demographic information, labour markets, health and health care information and household tourism activities. Population weights are available in the surveys for both households and individuals. To account for the different survey designs among the datasets, we use the adjusted survey weights provided by StatsSA.

### Variables definition and measurement

Health data are based on a short series of questions covering illnesses or injuries during the past 30 days prior to the survey, categories of disease/illness, dysfunctional disability lasting six months or more, categories of disabilities, whether an individual had access to medical aid coverage and the type of health care facility (public or private) where care would be sought in the event of illness. For a holistic outlook on health inequality, information related to health status in the two surveys were considered in our analysis.

Health status is measured by ill-health and disability. In the surveys, ill-health status is based on whether or not the respondent suffered from any illness or injury during the past month. Each respondent was asked whether he/she is limited in his/her daily activities, at home, at work or at school, because of a long-term physical, sensory, hearing, intellectual, or psychological condition, lasting six months or more. Positive responses are defined as disabled. Even though the GHS has some limitations, in that it does not contain explicit information on SAH and quality of health care, we use the information on ill-health/injury, which has been identified to be an important predictor of morbidity or mortality, especially in developing countries [64, 65].

Social determinants of health included in our analysis are based on WHO identified domains that influence pro-equity progress towards universal health care. Some of the domains include income and poverty, knowledge and education, housing and infrastructure, social protection, gender norms, and other individual/household factors [48]. Information collected in the GHS that is in line with the WHO identified domains on SDH, consistent in all surveys, and thus, used in our analysis includes: employment status; social grant recipient status; highest level of education completed (no schooling, less than diploma, diploma/certificate, university degree, and postgraduate degree); province and urban/rural setting; age; gender; race (black Africans, coloured, Asian/Indian and white), marital status (married, widow/widower, divorced/separated and single), and social grants/assistance receipt status.

Socio-economic variables such as education, social grants and employment were not just included in the analysis on the basis of WHO identified domains or data availability, but were included to capture the realities of changes in the socio-economic outlook of the country. For instance, since the emergence of democracy in South Africa, policy redressing all forms of socio-economic related inequalities were enacted, creating a more equitable national system. These policies improved access to education, social grants, employment, housing and health care, especially for the previously disadvantaged (non-white) groups. Over the post-apartheid period, there has been a relative increase in receipt of formal education and social grants by black Africans. In particular, the non-contributory old age pension and child grant have a strong racial dimension, with a considerable proportion of black Africans and coloureds as beneficiaries [66, 67].

Moreover, the GHS includes information on the ownership of household assets and services. We use that information to construct a wealth index, which serves as a proxy for our measure of socio-economic status; in the absence of data on household income. Following [68] 1, a wealth index was constructed in each of the survey years using the method of factor analysis (FA) 2 on a set of seven variables measuring relative wealth; source of drinking water, presence of electricity, land line/cellular phone, television set, radio, refrigerator and car. Thus, we were limited to wealth-related questions that were available in all surveys. Furthermore, this is our measure of socio-economic status.

### Empirical methods of estimating health inequality

#### Estimating a concentration index

As suggested earlier, we use the concentration index (*CI*) and decomposition of change in the concentration index for the analysis. The concentration index is employed because it presents an accurate picture of socio-economic inequalities in health, and has been used in a number of related studies [69–75].

For our empirical estimation, the standard concentration index is defined as twice the covariance between our health variable (*H*), e.g ill-health, and the ranking of socio-economic status (*S*) divided by the mean of the health variable, *μ* [69, 70, 76]:

1 $$ CI = \frac{2}{\mu}cov(H, S).  $$

It can also be written as :

2 $$ CI = \frac{2}{n\mu}\left[\sum\limits_{i=1}^{n}H_{i}S_{i}\right] - 1.  $$

where *μ* is the mean of *H*_*i*_; *S*_*i*_ is the fractional rank of the *i*th individual in the socio-economic groupings; *CI* is the concentration index which is the measure of relative inequality, such that doubling the health variable leaves *CI* unchanged. *CI* takes a value of zero when a health variable takes the same value among all individuals irrespective of their socio-economic status; *CI* is negative when a health variable is more concentrated among the poor than the better-off, and vice versa.

For ease of computation and generation of standard errors, from which statistical inferences can be made, the *CI* is specified as a regression:

3 $$ 2\sigma^{2}_{s}\left(\frac{H_{i}}{\mu}\right) = \alpha + \beta S_{i} +\sum\limits_{j} \beta_{j} X_{ji} + \upsilon_{i}.  $$

where $\sigma ^{2}_{s}$ is the variance of the fractional rank; *α* is the intercept; *β* is an estimate of the *CI*; *β*_*j*_ are the parameter vectors of the determinants *X*_*j*_; and *υ*_*i*_ is the error term.

#### Decomposing a change in concentration index

The concentration index of a health variable can be decomposed into the contributions of individual factors to its inequality, where each contribution is the product of the sensitivity of the health variable with respect to that factor and the degree of inequality in that factor [60].

Our decomposition analysis relies on modeling the linear relationship between a health variable of interest, *H*, and the contributions of the *j* determinants, *X*_*j*_:

4 $$ H = \alpha + \sum_{j} \beta_{j} X_{j}+ \upsilon.  $$

where *β*_*j*_ are the parameters’ coefficients of *X*_*j*_, and *υ* is the error term. By substituting (4) into (2), the overall concentration index (*CI*) can be rewritten as a linear combination of the concentration indices of the determinants, plus an error term, as expressed :

5 $$ C = \sum_{j} \left (\frac{\beta_{j}\bar X_{j}}{\mu}\right)C_{j}+ \frac{{GC}_{\upsilon}}{\mu}.  $$

where *μ* is the mean of health variable, *H*; $\bar X_{j}$ is the mean of each *j* determinant; *C*_*j*_ is the concentration index for the *jth* determinants, calculated from (2) by replacing the health variable (*H*_*i*_) with the determinant (*X*_*j*_) (defined analogously to *C*); *GC*_*υ*_ is the generalised concentration index for the error term (*υ*), and *C* is made up of two components (5). The first is the explained component, which is equal to a weighted sum of the concentration indices of the *j* regressors, where the weight is simply the elasticity of *H* with respect to $X_{j} \left (\eta _{j} = \beta _{j}\frac {\bar {X}_{j}}{\mu }\right)$. The second is the unexplained component, captured by the last term, $\frac {{GC}_{\upsilon }}{\mu }$; it is the inequality in health that cannot be explained by systematic variation across income groups in the *X*_*j*_.

As opposed to the cross-sectional decomposition above, it is possible to decompose health inequalities over time [60]. Specifically, one can unravel correlates of changes in health inequalities over time, by applying the Oaxaca-type decomposition [59].

To model the decomposition of change in the health inequalities across time, we apply the Oaxaca decomposition of change method to (5):

6 $$ \Delta C = \sum\limits_{j}\eta_{jt} \left (C_{jt}- C_{jt-1}\right) + \sum\limits_{j}C_{jt-1}\left (\eta_{jt} - \eta_{jt-1}\right) + \Delta \left (\frac {{GC}_{\upsilon t}}{\mu_{t}}\right).  $$

where *t* refers to time period and *Δ* denotes first differences. In (6), we weight the difference in concentration indices by the second period elasticity and weight the difference in elasticities by the first period concentration index. An alternative to (6) would be to weight the difference in concentration indices by the first period elasticity and weight the difference in elasticities by the second period concentration index as expressed in (7):

7 $$ \Delta C = \sum\limits_{j}\eta_{jt-1} \left (C_{jt}- C_{jt-1}\right) + \sum\limits_{j}C_{j}\left (\eta_{jt} - \eta_{jt-1}\right) + \Delta \left (\frac {{GC}_{\upsilon t}}{\mu_{t}}\right).  $$

As indicated earlier, this decomposition allows one to decompose the change in SES-related inequality in a health variable into changes in inequalities in its determinants, on one hand, and changes in the elasticities of the health variable with respect to these determinants, on the other hand. Our empirical estimation follows this approach in explaining changes in SES-related inequalities in health over time. Estimates were based on linear probability models (LPMs), which are appropriately weighted to the population and robust to heteroskedasticity. LPMs were employed due to the binary nature of the dependent health variables of interest.

One key assumption underlying the method is that there must be a consistent measure of socio-economic status, and population must be observed at least in two different points in time. In our analysis, the measure of socio-economic status is the wealth index, while we apply the method using three different time periods; 2004, 2010 and 2014. Data were analysed using Stata 14 [77].

## Results

### Data summary

Table 1 presents the data summaries by survey year. The results are presented in terms of the relative proportions of observations across the survey years. Compared with the 2004 and 2010 surveys, the 2014 survey contained fewer individuals aged less than 30 years, females and white. However, it contained more black Africans. The 2004 survey comprised more individuals below 30 years of age, females, coloured, white and single, while more married individuals are contained in the 2010 survey. Notably, the 2014 survey had more educated individuals with diplomas or certificates and an honours degree, but fewer people having no formal education. In comparison with 2004 and 2010, 2014 data is less rural, with fewer individuals living in the Eastern Cape, Northern Cape, Free State, Kwazulu-Natal and Limpopo, while more individuals are living in the relatively richer provinces of Western Cape and Gauteng. Moreover, social grant beneficiaries have greater coverage, while unemployment rates are lower in 2004 than in 2014. The results further suggest that the surveys contained similar proportions of Indians, as well as those who live in the Northwest and Mpumalanga provinces in 2004, 2010 and 2014.

**Table 1:** Summary statistics (mean) of the dependent and independent variables, GHS 2004, 2010 and 2014

Variable	2004	2010	2014
0 - 5 yrs	0.130	0.125	0.118
6 - 17 yrs	0.257	0.238	0.227
18 - 30 yrs	0.249	0.244	0.242
31 - 45 yrs	0.194	0.205	0.215
46 - 64 yrs	0.127	0.139	0.146
65 yrs +	0.043	0.048	0.053
Female	0.517	0.514	0.512
Black Africans	0.783	0.793	0.800
Coloured	0.091	0.090	0.090
Indian	0.025	0.025	0.025
White	0.101	0.091	0.085
Married	0.272	0.278	0.276
Single	0.728	0.720	0.722
No schooling	0.195	0.167	0.149
Less than diploma	0.740	0.753	0.746
Diploma/certificate	0.036	0.035	0.041
Honours degree	0.020	0.030	0.035
Postgraduate	0.004	0.003	0.004
Employed	0.308	0.286	0.306
Urban	0.563	0.614	0.638
Western Cape	0.107	0.111	0.114
Eastern Cape	0.135	0.128	0.124
Northern Cape	0.023	0.022	0.022
Free state	0.058	0.054	0.051
Kwazulu-Natal	0.202	0.199	0.197
Northwest	0.068	0.068	0.068
Gauteng	0.224	0.235	0.242
Mpumalanga	0.078	0.078	0.078
Limpopo	0.106	0.105	0.104
Grant recipients	0.098	0.275	0.290
Illness	0.113	0.113	0.097
Disability	0.026	0.080	0.031
Medical aid coverage	0.156	0.180	0.179
Public health facility	0.751	0.730	0.734
Private health facility	0.248	0.253	0.262
	Mean	Mean	
Observations	97,036	95,764	92, 445

Estimates are weighted to the population using the sample weights

Compared with 2004 and 2010, health status in 2014 is better, as a lower share of individuals reported ill-health. On the contrary, there is a sharp increase in disability reports in 2010. Similarly, medical aid coverage rates are proportionately higher in 2010, than in 2004 and 2014. However, fewer individuals prefer to utilise public health care when ill in 2010, while proportionally more individuals would prefer private health care in 2014, than in previous studied periods.

### Health concentration indices for 2004, 2010 and 2014

Table 2 presents the health concentration indices for 2004, 2010 and 2014 (estimates are from (3)). The results show the relative changes in health inequalities over the different time periods. Ill-health is more concentrated among the poor in 2010, but more concentrated among the better-off in 2004 and 2014; however, the result is not significant at conventional levels in 2014. Similarly, disability is somewhat more concentrated among the poor in 2004 and 2010, while it is more concentrated among the better-off in 2014, although the results are not statistically significant in 2004 and 2010.

**Table 2:** Health Concentration Indices and Social Determinants for 2004, 2010 and 2014, GHS South Africa

	III-health status						Disability					
	2004		2010		2014		2004		2010		2014	
	*Coeff*	*se*	*Coeff*	*se*	*Coeff*	*se*	*Coeff*	*se*	*Coeff*	*se*	*Coeff*	*se*
*CI*	0.018*	(0.008)	-0.017*	(0.008)	0.009	(0.010)	-0.000	(0.016)	-0.004	(0.008)	0.033*	(0.014)
0 - 5 yrs	-0.136***	(0.015)	-0.156***	(0.013)	0.025	(0.013)	-0.377***	(0.036)	0.351***	(0.018)	-0.673***	(0.039)
6 - 17 yrs	-0.211***	(0.013)	-0.207***	(0.012)	-0.054***	(0.011)	-0.038	(0.032)	-0.181***	(0.013)	-0.432***	(0.029)
18 - 30 yrs	-0.207***	(0.013)	-0.187***	(0.013)	-0.067***	(0.012)	0.059	(0.033)	-0.129***	(0.014)	-0.327***	(0.031)
31 - 45 yrs	-0.126***	(0.013)	-0.132***	(0.013)	-0.042***	(0.012)	0.247***	(0.034)	-0.097***	(0.014)	-0.269***	(0.030)
46 - 64 yrs	-0.047***	(0.013)	-0.052***	(0.013)	-0.028*	(0.012)	0.384***	(0.036)	-0.067***	(0.014)	-0.211***	(0.030)
Female	0.019***	(0.004)	0.028***	(0.004)	0.030***	(0.004)	-0.129***	(0.009)	-0.012**	(0.004)	-0.004	(0.007)
Black African	-0.014	(0.008)	-0.010	(0.008)	-0.003	(0.008)	-0.090***	(0.019)	-0.010	(0.008)	-0.016	(0.014)
Indian/Asians	-0.001	(0.015)	0.006	(0.014)	-0.045**	(0.015)	-0.001	(0.032)	-0.003	(0.015)	-0.008	(0.024)
White	-0.025*	(0.011)	-0.001	(0.011)	0.053***	(0.012)	-0.025	(0.021)	0.002	(0.010)	0.023	(0.018)
Single	0.012*	(0.006)	0.014*	(0.006)	-0.000	(0.006)	0.171***	(0.014)	0.029***	(0.004)	0.074***	(0.009)
No Schl.	0.031	(0.026)	0.064***	(0.016)	0.046**	(0.017)	0.120	(0.065)	0.070***	(0.021)	0.179***	(0.041)
Less Diploma	0.008	(0.025)	0.042**	(0.014)	-0.006	(0.014)	-0.213***	(0.059)	-0.075***	(0.017)	-0.166***	(0.028)
Diploma Cert.	0.017	(0.027)	0.051**	(0.019)	0.007	(0.017)	-0.237***	(0.060)	-0.063***	(0.019)	-0.184***	(0.030)
Honours degree	0.001	(0.031)	0.046*	(0.020)	0.007	(0.019)	-0.278***	(0.058)	-0.069***	(0.018)	-0.201***	(0.030)
Employed	-0.003	(0.005)	-0.013*	(0.005)	0.003	(0.006)	-0.229***	(0.010)	-0.035***	(0.004)	-0.081***	(0.008)
Urban	0.008	(0.005)	0.039***	(0.005)	0.036***	(0.005)	0.004	(0.010)	0.007	(0.005)	0.009	(0.009)
Western Cape	-0.102***	(0.010)	-0.021*	(0.009)	-0.044***	(0.010)	0.048	(0.025)	-0.056***	(0.010)	-0.028	(0.018)
Eastern Cape	-0.055***	(0.009)	-0.054***	(0.008)	0.030**	(0.010)	0.012	(0.017)	-0.002	(0.010)	-0.041*	(0.017)
Northern Cape	-0.085***	(0.011)	0.026*	(0.010)	0.015	(0.011)	-0.019	(0.023)	0.030**	(0.011)	0.044*	(0.023)
KwaZulu - Natal	-0.114***	(0.009)	-0.035***	(0.008)	-0.043***	(0.009)	-0.002	(0.017)	-0.050***	(0.009)	-0.072***	(0.016)
North West	-0.009	(0.010)	0.000	(0.009)	0.009	(0.011)	0.026	(0.020)	-0.024*	(0.010)	0.038	(0.020)
Gauteng	-0.054***	(0.009)	-0.019*	(0.008)	0.029**	(0.010)	-0.001	(0.016)	-0.105***	(0.008)	-0.054***	(0.015)
Mpumalanga	-0.012	(0.010)	-0.014	(0.008)	0.021*	(0.010)	0.018	(0.019)	-0.084***	(0.009)	-0.056**	(0.017)
Limpopo	-0.061***	(0.009)	0.010	(0.009)	-0.023*	(0.010)	-0.038*	(0.018)	0.017	(0.010)	-0.065***	(0.018)
Grant recipients	0.093***	(0.008)	0.020***	(0.005)	-0.004	(0.007)	0.557***	(0.028)	0.141***	(0.007)	0.291***	(0.015)
Constant	0.346***	(0.031)	0.257***	(0.022)	0.162***	(0.021)	0.286***	(0.072)	0.285***	(0.025)	0.543***	(0.045)
Adjusted *R*^2^	0.03		0.02		0.02		0.07		0.19		0.05	
Observation	97,036		95,764		92,445		97,036		95,764		92,445	

Standard errors reported in parentheses

* *p*<0.05, ** *p*<0.01, *** *p*<0.001

Ill-health concentration indices for 2004 and 2010 were 0.018 and -0.017, respectively, indicating a concentration of ill-health amongst the poor over time, and an increase in inequality between 2004 and 2010. Similarly, disability concentration indices for 2004 and 2010 were -0.000 and -0.004, respectively, suggesting that disability was more concentrated among the poor in each, and a worsening in inequality between the time period. Between 2004 and 2010, changes in ill-health and disability concentration indices were -0.035 and -0.004, respectively. These changes are pro-poor and widened over that time period. On the contrary, the concentration indices for ill-health and disability changed by 0.026 and 0.037 over the period 2010 and 2014, suggesting that they become more concentrated among the better-off. In what follows (see Table 3), we present the results of the social determinants that contribute to the observed changes in the health concentration indices. Each row in Table 3 was estimated based on the calculations from (6). In presenting the results, we emphasise variables whose socio-economic inequalities have consistently reduced health inequalities over the periods.

**Table 3:** Oaxaca-type decomposition of change in the health inequalities, 2004–2010 and 2010–2014

	2004–2010						2010–2014					
	Ill-health status			Disability			Ill-health status			Disability		
	*Δ* *C*	*se*	%	*Δ* *C*	*se*	%	*Δ* *C*	*se*	%	*Δ* *C*	*se*	%
0 - 6 yrs	-0.004	0.008	-9.4	-0.046	0.008	191.1	-0.013	0.008	-92.9	0.059	0.009	311.2
6 - 17 yrs	-0.001	0.009	-1.9	0.006	0.008	-26.4	-0.006	0.009	-43.1	0.023	0.010	122.3
18 - 30 yrs	0.002	0.010	4.0	0.008	0.009	-34.3	-0.006	0.010	-42.9	0.014	0.010	72.9
31 - 45 yrs	0.004	0.011	10.5	-0.021	0.009	86.4	0.005	0.010	33.6	-0.006	0.010	-30.4
46 - 64 yrs	0.003	0.013	7.4	-0.039	0.010	159.7	0.002	0.012	12.2	-0.018	0.011	-96.3
Black African	-0.006	0.003	-14.3	-0.055	0.002	227.5	-0.001	0.003	-11.1	0.007	0.002	38.8
Indian	0.000	0.016	-0.9	0.000	0.007	0.5	-0.004	0.016	-32.6	0.000	0.007	-0.5
White	0.009	0.018	23.3	0.012	0.008	-49.9	0.020	0.017	146.9	0.009	0.008	47.5
Single	0.019	0.018	48.6	0.032	0.007	-131.3	0.002	0.019	18.0	-0.013	0.008	-70.9
No schooling	0.001	0.231	1.5	0.013	0.236	-53.3	0.004	0.033	31.0	-0.010	0.021	-54.4
Less than diploma	0.000	0.019	-1.0	-0.003	0.020	11.3	0.002	0.013	15.2	0.021	0.014	108.8
Diploma certificate	-0.001	0.018	-3.7	0.022	0.019	-91.5	-0.003	0.011	-20.7	-0.015	0.012	-77.9
Honours degree	0.001	0.020	3.8	0.017	0.019	-69.6	-0.003	0.014	-21.5	-0.019	0.012	-98.9
Employed	-0.002	0.040	-4.1	0.063	0.025	-261.5	0.004	0.036	26.0	-0.012	0.020	-62.6
Urban	0.018	0.004	47.8	0.001	0.002	-6.1	0.001	0.004	8.8	0.007	0.002	35.7
Western Cape	0.014	0.005	35.3	-0.018	0.003	75.8	-0.005	0.005	-40.7	0.004	0.003	22.1
Eastern Cape	-0.002	0.011	-4.0	0.003	0.006	-11.7	-0.012	0.011	-92.4	0.006	0.006	33.8
Northern cape	0.000	0.008	1.0	0.000	0.006	-0.5	0.000	0.009	-0.1	0.000	0.007	0.6
Kwazulu-Natal	-0.011	0.010	-27.6	0.007	0.006	-29.7	0.002	0.010	15.9	0.004	0.007	23.4
North West	-0.011	0.010	-27.6	0.001	0.006	-5.4	0.000	0.010	-2.7	-0.003	0.007	-14.5
Gauteng	0.009	0.008	24.0	-0.032	0.005	133.3	0.015	0.008	114.5	0.013	0.006	66.6
Mpumalanga	0.000	0.009	0.6	0.002	0.005	-8.6	-0.001	0.009	-9.8	-0.001	0.006	-4.0
Limpopo	-0.008	0.010	-20.1	-0.007	0.005	28.7	0.005	0.010	35.8	0.013	0.006	67.8
Grant recipient	0.002	0.009	5.7	0.008	0.006	-32.3	0.007	0.010	54.1	-0.064	0.006	-341.8
Female	0.000	0.006	1.1	0.000	0.005	-2.0	0.000	0.005	-1.8	0.000	0.004	0.7
Total	0.039			-0.024			0.013			0.019		

The results further suggest that, in all the survey years, ill-health is less concentrated among the relatively younger individuals, when compared with those aged above 65 years. Similarly, those living in the Western Cape and KwaZulu-Natal provinces report illness less often. Furthermore, those who are relatively educated and employed are less likely to report disability, while the opposite was observed for females and those without formal education. We find disability to be less concentrated among those who are educated and employed in 2004, 2010 and 2014. Disability is also found to be less concentrated among residents of relatively urban provinces. However, single individuals, those without formal education and social grant recipients are more likely to suffer from disability; such a result is expected, since one of the social grants is a disability grant.

### Decomposition result

In Table 3, we present the Oaxaca decomposition results for the health indicators, separately, for 2004–2010 and 2010–2014, respectively. These results show the extent to which inequalities in the health indicators are due to changes in socio-economic inequalities in their associated social determinants. While interpreting these results, one needs to bear in mind that our proxy for socio-economic status is wealth, and ill-health and disability inequalities relatively worsened (became more pro-poor) over the period 2004–2010, while the inequalities narrowed (became more pro-rich) between 2010–2014. Each row in Table 3 is thus interpreted in terms of its socio-economic contribution to the observed changes in the health inequalities, over time. A negative sign on a variable denotes that the contribution of the socio-economic inequality in that variable worsens health inequalities over the period 2004–2010, while a positive sign indicates that socio-economic inequality, probably reduced, and thus such variable contributes to lessen health inequalities over the period 2010–2014.

With respect to ill-health inequalities, we find that age differences matter in explaining changing ill-health inequalities over the two time periods (2004–2010 and 2010–2014). Between 2004–2010, socio-economic inequalities among those who are below the age of 17 years, relative to older individuals, contribute to ill-health inequality. One can infer that children below the ages of 17 years lived in relatively poor households that likely had characteristics that increased the likelihood of them falling ill. We, however, observe the opposite result for the period 2010–2014. In contrast to 2004–2010 results, socio-economic inequalities among those who were aged above 30 years account for widening ill-health inequalities over the period 2010–2014. This signals a relative improvement in socio-economic status among individuals in this age category.

While socio-economic inequalities among whites explain considerable reduction in ill-health inequality between 2010 and 2014, socio-economic inequalities among black Africans, relative to other population groups, worsen ill-health inequalities over the period 2004–2010. Also, ill-health inequalities over the period 2004–2010 were caused by socio-economic inequalities relating to educational attainment and employment status, as socio-economic inequalities among those who hold a diploma/certificate and those who are employed explain ill-health inequalities. On the other hand, socio-economic inequalities among singles and those with little or no formal education contribute to changing ill-health inequalities over the period 2010–2014.

Notably, socio-economic inequalities relating to employment status, among those living in KwaZulu-Natal, North West and Limpopo, explain an improvement in ill-health inequalities in both time periods. For instance, the results suggest that socio-economic inequalities between the employed and unemployed worsened ill-health inequality over the period 2004–2010, while an improvement in ill-health inequalities, between 2010–2014, was caused by reduced socio-economic inequalities relating to employment status. On the other, ill-health inequalities worsened, based on increased socio-economic inequalities among those living in Eastern Cape.

Similar to ill-health results, we observe that age differences also matters in explaining differences in disability inequalities in the two time periods. Between 2010–2014, socio-economic inequalities among those who are below the age of 30 years contribute to changes in disability inequality. By contrast, socio-economic inequalities among those above the age of 30 years account for disability inequalities over the the period 2004–2010. Over the period 2010–2014, inequalities among those who are at least diploma certificate holders, employed, living in Free state and recipients of social grants explain disability inequality. Over the two time periods, socio-economic inequalities among black Africans, those having less than diploma, living in Western Cape, Gauteng and Limpopo provinces explain changing disability inequalities. For instance, while socio-economic inequalities among black Africans worsen disability inequalities between 2004–2010, disability inequalities were enhanced (became more pro-rich) by reduced socio-economic inequalities among black Africans.

Overall, taking the changes in all the determinants of the health variables into account, the results suggest that the bulk of changes in ill-health inequalities, over the two time periods, are consistently explained by socio-economic inequalities relating to employment status and provincial differences, which narrowed considerably over the studied periods. Relatedly, disability inequalities are largely explained by shrinking socio-economic inequalities within racial groups, educational attainment and provincial differences.

## Discussion

This paper examines and compares the relative changes in socio-economic inequalities in health status, mainly ill-health status and disability, between 2004–2010 and 2010–2014 in post-apartheid South Africa, linking those changes to changes in social determinants. It emerged that socio-economic inequalities exist in the health variables and the inequalities widened over the period 2004–2010, but narrowed over the period 2010–2014. Socio-economic inequalities, in favour of the better-off, also exist in disability over time. Over time, relatively richer individuals suffer from disability compared to their poorer counterparts. These findings resonate with existing evidence from similar studies carried out in developed and developing countries [14, 16, 19, 73, 78–81], which suggest that rates of self-assessed poor health could be substantially higher among lower socio-economic groups, and that the magnitude of inequalities in SAH varies substantially across income distributions. In 17 European countries, almost no country consistently experienced a significant decline in either absolute or relative inequalities over time [7].

Also, in line with our findings, poor self-reported health is higher among lower socio-economic groups in South Africa [50, 51, 55]. Socio-economic inequalities in self-reported health tend to be greater among lower socio-economic groups [50]. Basically, pro-poor inequalities in ill-health status and disability found in this study are consistent with earlier studies, which found ill-health and disability to be more concentrated among the poor [48, 50, 51]. In addition, our analyses, which cover a more recent time period find a shift towards a pro-rich inequalities in ill-health and disability, over time.

More pertinently, our findings show that changes in inequalities in SDH play an important role in explaining changes in health over the two time periods, 2004–2010 and 2010–2014. Specifically, we find that social drivers of changing inequalities in ill-health status are related to changing socio-economic inequalities within racial groups, especially among black Africans, which widened considerably over time. Socio-economic inequalities in employment, educational attainment relating to diploma/certificate also explain inequalities in ill-health status over time. Moreover, socio-economic inequalities among those living in the Eastern Cape and KwaZulu-Natal consistently explain ill-health inequalities over the studied periods. Notably, disability inequalities are uniformly explained by inequalities in educational attainment, particularly having less than diploma, though such inequalities narrowed substantially. Socio-economic inequalities among those residing in the Western Cape and Gauteng also consistently explained disability inequalities over the studied periods.

“Causes” of socio-economic inequalities in health include socio-economic status, education, urbanisation, geographics and housing. They are important factors that determine health inequity in most countries, especially in developing countries [5, 19, 73, 82, 83]. In line with the preceding studies, socio-economic factors are significant in explaining health inequality over time [7]. In South Africa, social protection and employment, knowledge and education, housing and infrastructure contribute to disparities in good SAH [48]. Relatedly, we find race, education and geography/provincial location to be key social drivers of health inequalities.

Changes in socio-economic inequalities in health can be explained by changes in inequalities in social determinants, namely education, income, housing and residential locations. Thus, social factors have an important influence in determining health status and explaining observed health inequalities over time[10, 19, 52, 84–88]. In addition to this study shedding more light on changing health inequalities in South Africa, the social determinants-health inequalities nexus unraveled in this study further affirm and complement other related studies which have examined social gradients in health in South Africa [21, 23, 33, 48, 50–57].

Overall, socio-economic inequalities relating to racial groups, geography/residential locations and educational attainment and employment status are found to be crucial in explaining inequalities in health over time. Although we did not establish any direct causal link, this finding, nonetheless, highlights the importance of the identified social factors in reducing health inequalities. Considering South Africa’s past history of Apartheid and systemic racial discrimination, it is not too surprising that trends in inequalities in the identified social factors remain apparent in the analysis of SDH-health inequalities link. Plausibly, more concerted policy action and interventions to tackle further inequalities in social factors could help push the country towards a more equitable health care system. This might require new social-oriented policies emphasizing further reductions in inter- and intra- racial group inequalities and unemployment, improved access to education, especially for the previously disadvantaged groups, and more concrete and sustainable development programmes at provincial levels, while strengthening the existing ones that have proved to be somewhat effective. Available evidence suggests that, over the post-apartheid period, South Africa has maintained a reasonably equitable racial and gender balance in education and social grant receipt [67, 89, 90]. In particular, the non-contributory and unconditional grants have a strong racial dimension, with a sizeable proportion of previously disadvantaged groups as beneficiaries [91]. Despite all these polices and programmes, there remains a social gradient in key SDH in South Africa. Thus, there is more to be done to strengthen policy and institutions to further tackle socio-economic related health inequalities.

## Conclusion

Using nationally representative data from the 2004, 2010 and 2014 General Household Surveys (GHSs), this research uncovers the relative changes in health inequalities over the second decade of post-apartheid South Africa. It also provides an explanation for changes in the social determinants of health that account for disparities in health over that period. The health indicators considered in the analysis include ill-health and disability. The concentration index regression model and the Oaxaca-type decomposition of change in the concentration index were employed to achieve the stated objectives.

Notably, socio-economic related inequalities in health have been identified as one of the greatest challenges to public health in South Africa. Our findings show that a number of social factors, including education, employment status, provincial and racial differences need to be addressed in order to further tackle the avoidable and widely considered unacceptable socio-economic health inequalities in South African society. Evidence from this study explicitly supports the theories and views that the causes of social inequalities in health are multiple and inter-related. Actions to further tackle these, thus, need to be interconnected, intersectoral and across multiple intervention levels.

This study, therefore, recommends policies that are explicitly intersectoral in nature, which will entail multiple social interventions. The recommendations are aptly situated within the context of research on the typology of actions to tackle social inequalities in health [1, 92–94]. Tackling health inequalities through policies and interventions which are based on the underlying theory of how the action is expected to bring about the desired change [1]. The author proposes that the common interventions tend to fall into one of four main categories, which include: strengthening individuals, strengthening communities, improving living and working conditions and associated access to essential services, and promoting healthy macro-policies. Our research supports that view.

## Appendix A

### Mathematical derivation of wealth index

In mathematical terms, from an initial set of *j* correlated variables, FA creates uncorrelated indices or components where each component is a linear weighted combination of the initial variables. Following [68], given *j* random variables *x*_1_,....., *x*_*j*_, the FA wealth index can simply be represented as follows:

8 $$ {FA}_{1} = \lambda_{11} x_{1}+ \lambda_{21} x_{2}+ \lambda_{31} x_{3}+..........+ \lambda_{j1} x_{j}.  $$

In the case where the wealth variables, *x*_*i*_{*i*=1....*j*}, are not standardised; to a mean of zero and variance of one, they are first standardized such that the equation for the first principal component is given by:

9 $$\begin{array}{*{20}l} {FA}_{1} & = \lambda_{11} \left (\frac {x_{1} - \bar x_{1}} {s_{1}}\right) + \lambda_{21} \left (\frac {x_{2} - \bar x_{2}} {s_{2}}\right)  \\ &\quad+ \lambda_{31} \left (\frac {x_{3} - \bar x_{3}} {s_{3}}\right) +.....+ \lambda_{j1} \left (\frac {x_{j} - \bar x_{j}} {s_{j}}\right) \end{array} $$

10 $$\begin{array}{*{20}l} & = \frac{\lambda_{11}}{s_{1}}x_{1} + \frac{\lambda_{21}}{s_{2}}x_{2} + \frac{\lambda_{31}}{s_{3}}x_{3} +..........+ \frac{\lambda_{j1}}{s_{j}}x_{j} - a. \end{array} $$

The coefficients *λ*_*j*1_ represent the elements of the eignevector *λ*_1_ associated with the largest eigenvalue of the correlation matrix of the *x*_*i*_ variables. The constant *a* is the weighted sum of the means, which ensure that *FA*_1_ has a mean of zero. In a more generic term, the wealth index, *FA*_*i*_ for individual *j* can thus be defined as:

11 $$ {FA}_{1} = \sum\limits_{j}\left(\lambda_{j}\frac {\left({x_{j} - \bar x_{j}}\right)}{s_{j}}\right).  $$

## Abbreviations

GHS: General Household Survey
SAH: Self assessed health
SDH: Social determinants of health

## Footnotes

1 Several articles have validated the use of wealth indices against external criteria. They conclude that they are good proxies for income differences [95–98]. A detailed description of the construction of the wealth index is contained in “Appendix A” section.

2 Factor analysis (FA) is a multivariate statistical technique used to reduce the number of variables in a data set into a smaller number of ’dimensions’ [99, 100].
